# MiR396 is involved in plant response to vernalization and flower development in *Agrostis stolonifera*

**DOI:** 10.1038/s41438-020-00394-x

**Published:** 2020-11-01

**Authors:** Shuangrong Yuan, Zhigang Li, Ning Yuan, Qian Hu, Man Zhou, Junming Zhao, Dayong Li, Hong Luo

**Affiliations:** 1 Department of Genetics and Biochemistry, Clemson University, 110 Biosystems Research Complex, Clemson, SC 29634 USA; 2 Department of Grassland Science, Sichuan Agricultural University, 611130 Chengdu, Sichuan China; 3 Beijing Vegetable Research Center, Beijing Academy of Agriculture and forestry Science, 100097 Beijing, China

**Keywords:** Non-coding RNAs, Transgenic organisms

## Abstract

MicroRNA396 (miR396) has been demonstrated to regulate flower development by targeting *growth-regulating factor*s (*GRFs*) in annual species. However, its role in perennial grasses and its potential involvement in flowering time control remain unexplored. Here we report that overexpression of miR396 in a perennial species, creeping bentgrass (*Agrostis stolonifera* L.), alters flower development. Most significantly, transgenic (TG) plants bypass the vernalization requirement for flowering. Gene expression analysis reveals that miR396 is induced by long-day (LD) photoperiod and vernalization. Further study identifies *VRN1*, *VRN2*, and *VRN3* homologs whose expression patterns in wild-type (WT) plants are similar to those observed in wheat and barley during transition from short-day (SD) to LD, and SD to cold conditions. However, compared to WT controls, TG plants overexpressing miR396 exhibit significantly enhanced *VRN1* and *VRN3* expression, but repressed *VRN2* expression under SD to LD conditions without vernalization, which might be associated with modified expression of methyltransferase genes. Collectively, our results unveil a potentially novel mechanism by which miR396 suppresses the vernalization requirement for flowering which might be related to the epigenetic regulation of *VRN* genes and provide important new insight into critical roles of a miRNA in regulating vernalization-mediated transition from vegetative to reproductive growth in monocots.

## Introduction

Flowering is a crucial phase to determine plant reproductive success. Many crop species require prolonged exposure to the winter cold or vernalization to promote their vegetative-to-reproductive stage transition. However, vernalization and flowering time largely limit crop growth regions due to varied winter temperature from place to place and from year to year. Therefore, it is highly valuable to study the molecular mechanisms of vernalization and develop new strategies to control flowering time.

The molecular mechanisms and control of vernalization have been well studied in *Arabidopsis*. FLOWERING LOCUS C (FLC), a MADS box protein, serves as a repressor of flowering in *Arabidopsis*^1^, suppressing flowering promoters *FLOWERING LOCUS T* (*FT*), *FLOWERING LOCUS D* (*FD*), and *SUPPRESSOR OF OVEREXPRESSION OF CONSTANS 1* (*SOC1*) in leaves and meristems, respectively^2^. Interestingly, genes involved in vernalization are different between *Arabidopsis* and the cereals wheat (*Triticum aestivum* L.) and barley (*Hordeum vulgare* L.). Thus far, a molecular framework for flowering time control in response to vernalization has been established in wheat and barley through identification and characterization of *VERNALIZAITON1* (*VRN1*), *VRN2*, and *VRN3*^3–5^. *VRN1* is a MADS-box transcription factor gene, which regulates the vegetative to reproductive phase transition^6^. *VRN2*, a flowering repressor, encodes a CCT domain and zinc finger containing protein not related to FLC^7^. *VRN3*, the ortholog of *FT* in wheat and barley, has a similar mechanism in promoting flowering as *FT* does in *Arabidopsis*^8^. Before vernalization, high levels of *VRN2* repress the florigen *VRN3* to prevent flowering^7^. During cold exposure, *VRN1* is activated through chromatin modifications, including a decrease in H3K27 methylation and an increase in H3K4 methylation^9^. High levels of *VRN1* down-regulate *VRN2* during and after vernalization in leaves and meristems, which facilitates the accumulation of *VRN3* in leaves by LD photoperiod after vernalization^3,10^. *VRN3* then moves to the shoot apex to maintain the high levels of *VRN1* and induces flowering^3^. Recently, the molecular mechanisms of vernalization response have been gradually uncovered in *Brachypodium distachyon*, a small temperate grass in the subfamily Pooideae that also includes wheat and barley. In Brachypodium, *VRN1* and *VRN3*/*FT* have similar mechanisms as those in wheat and barley, whereas *VRN2* is induced during prolonged cold^11^, indicating that although within the same subfamily, the molecular mechanism of plant vernalization response is not conserved.

Accumulating evidence demonstrates that microRNAs (miRNAs) are involved in flower development, including floral organ identity and polarity, floral organ size and shape, inflorescence development, male and female fertility, and flowering time control^12,13^. However, there has been no evidence suggesting that miRNAs are directly involved in the vernalization pathway.

MiR396 is an evolutionarily conserved miRNA that targets transcription factors, GROWTH-REGULATING FACTORS (GRFs), involved in plant development during both vegetative and reproductive stages^14,15^. Morphologically, transgenic plants (TG) constitutively expressing miR396 displayed shorter stature and narrower leaves than wild type (WT) controls in *Arabidopsis* and tobacco^16,17^. Moreover, miR396 also controls plant development in the reproductive stage. Overexpression of miR396 resulted in stigmatoid anthers or a fasciated style with multiple stigma structures in transgenic tobacco^18^, pistil abnormalities in transgenic *Arabidopsis*^19^, or open husks, long lemmas and increased leaf angle in transgenic rice^20,21^. Our recent work characterizing miR396 revealed its involvement in the regulation of plant response to abiotic stress. Overexpression of a rice miR396 gene, *Os-miR396c* in a perennial species, creeping bentgrass (*Agrostis stolonifera* L.) not only altered leaf morphology and tillering, but also led to enhanced plant resistance to salinity in transgenics^22^. However, the impact of miR396 on floral organ development in perennial species remains elusive. In addition, there has been no report indicating the involvement of miR396 in flowering time control or vernalization pathway in any plant species.

To investigate the role of miR396 in flower development in perennial species, we further characterized miR396-overexpressing creeping bentgrass plants. Our data indicate that transgenic plants exhibit stamen defects and bypass vernalization to flower. Gene expression and RNA-seq analyses demonstrate that miR396-mediated flowering time control requires the coordinated regulation of multiple factors, including miR396-GRF module, *VRN* genes, and other regulatory elements. Our results unveil a potentially novel mechanism by which miR396 suppresses the vernalization requirement for flowering and provide important new insights into critical roles of a miRNA in the regulation of vernalization-mediated transition from vegetative to reproductive growth in monocot species.

## Materials and methods

### Plant growth

Transgenic creeping bentgrass plants constitutively expressing the rice *Osa-miR396c* gene were produced by *Agrobacterium*-mediated plant transformation as described in Yuan et al.^22^. Wild type (WT) and the regenerated transgenic (TG) creeping bentgrass plants were clonally propagated and grown in plastic pots (15 × 10.5 cm, Dillen Products) filled with commercial nutrient-rich soil (3-B Mix, Fafard). The plants were fertilized weekly with 0.2 g/L 20:10:20 water-soluble fertilizer (Peat-Lite Special; The Scotts Company) and were maintained in a growth room with SD light regime (14 h of light/10 h of dark). Temperatures in the SD growth room were 25 °C during the light period and 17 °C during the dark period with 350–450 μmol m^−2^ s^−1^ light intensity. The conditions for the LD growth room were the same as the SD growth room, except that the light regime is 16 h of light/8 h of dark. The vernalization treatment was performed in a cold room at 5 °C in an 8-h-ligh/16-h-dark photoperiod. Plants were grown under fluorescent bulbs and the light intensity was 100–150 μmol m^−2^ s^−1^ at plant level. WT and TG plants were propagated at the same time and from the same number of tillers to ensure that they were at the same developmental stage before LD induction and vernalization treatment. We rotated plants every other day to minimize the difference of light intensities on plant growth within each growth room.

### Microscopic observations

Spikelets, floralets, anthers, and pollens of WT and TG plants were observed under a stereo microscope (MEIJI EM-5 and MEIJI EMZ-5TR) and photographed. Spikelets and florets of vernalized WT and TG plants at 6th and 8th week after LD induction were detached for observation. WT and TG anthers were detached and observed 1 day before dehiscence. To compare WT and TG pollen viability, pollen was taken out of WT and TG anthers during the highest pollen viability rate of creeping bentgrass (9:00 a.m.) and stained with 2% (m/v) potassium iodide for microscopic observation.

### Plant RNA isolation and expression analysis

Plant RNA isolation, semi-quantitative, and real-time RT-PCR analyses were conducted with previously published protocol^22^. The expression of every candidate gene was analyzed in three biological replicates. Data from a representative replicate of the semi-quantitative RT-PCR results were presented.

### Phylogenetic analysis

The sequence alignments were performed by using the complete amino acid sequences of the three creeping bentgrass proteins, AsVRN1, AsVRN2, and AsVRN3 identified in this study and their orthologs in rice, *Arabidopsis*, wheat, barley, and *Brachypodium* based on previous study^11,23^. Phylogenetic trees were generated from the aligned sequences by using the neighbor jointing method in MEGA 6^24^. The confidence values for the nodes were derived from 1000 bootstrap replicates.

### RNA-seq data analysis

WT and TG plants cDNA library preparation, Illumina sequencing, differential gene expression analysis, GO enrichment analysis, and RT-qPCR analysis were performed and described elsewhere^22^. All primers used for quantitative and semi-quantitative RT-PCR analysis are listed in Table S1.

## Results

### Overexpression of miR396c eliminates vernalization, but not long-day requirements for flowering

We have previously generated transgenic creeping bentgrass plants constitutively expressing a rice miR396c gene and three representative transgenic lines, TG2, TG7, and TG14 were analyzed to study miR396-mediated plant development and stress response^22^. The same transgenic lines were further characterized in the present research to investigate miR396-mediated flower development in perennial grasses.

**Fig. 1:**
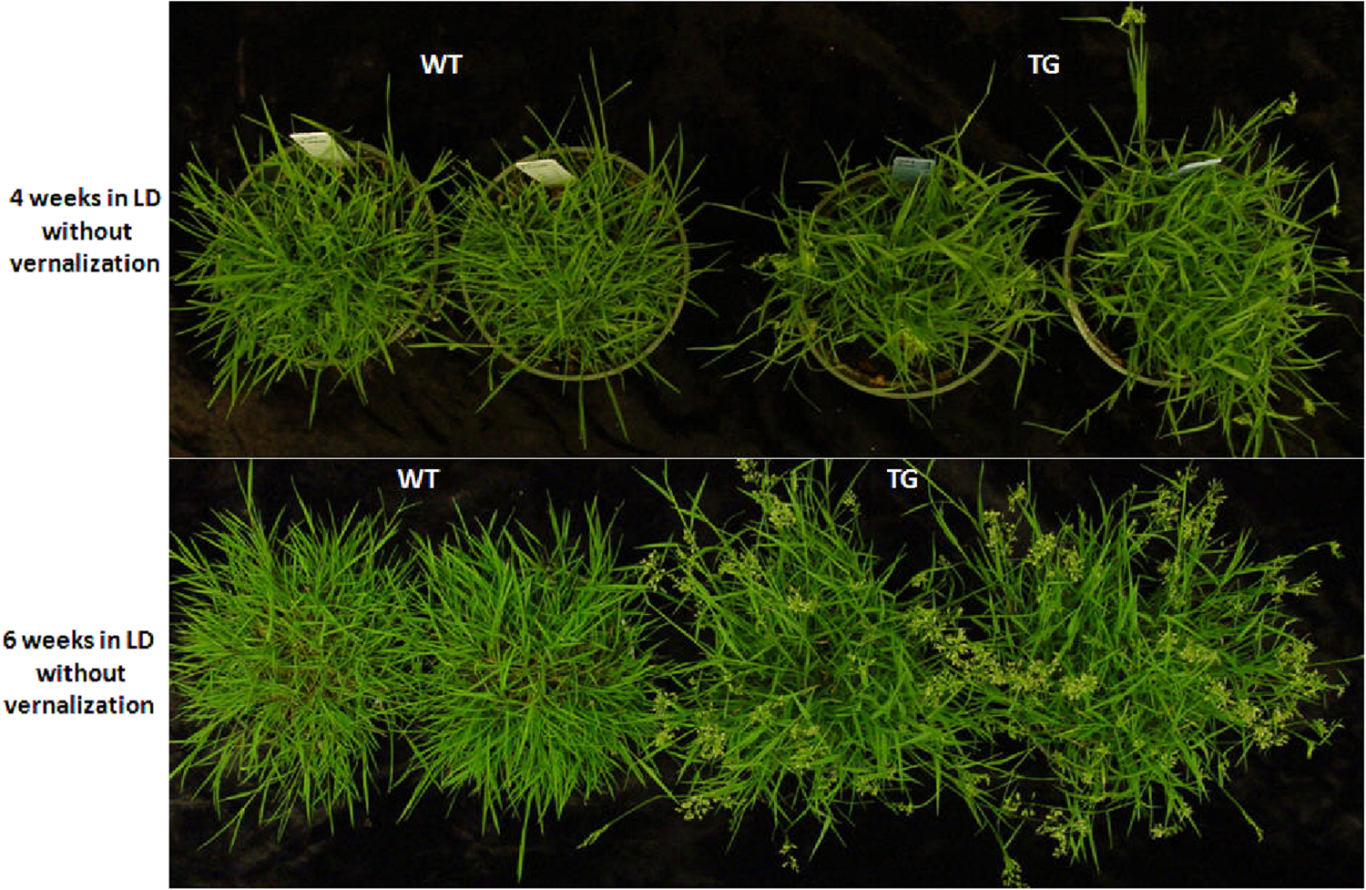
*Osa-miR396c* transgenic creeping bentgrass flowers without vernalization. Performance of WT and TG plants under 16-h photoperiod induction for 4 and 6 weeks without vernalization

**Fig. 2:**
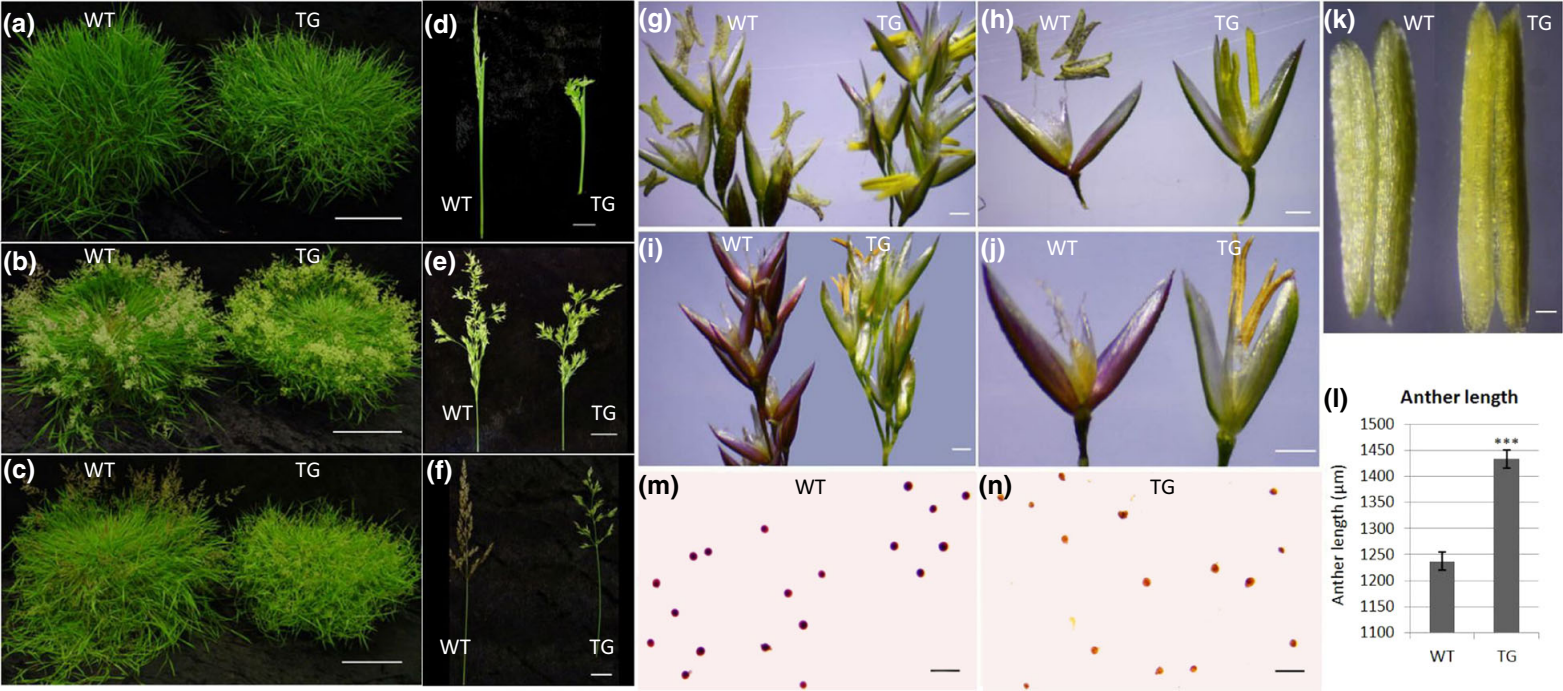
Flower development in WT and TG plants. Vernalized WT and TG plants under LD conditions for **a** 4 weeks, **b** 6 weeks, and **c** 8 weeks. Scale bar, 10 cm. WT and TG spikelets after LD induction for **d** 4 weeks, **e** 6 weeks, and **f** 8 weeks. Scale bar, 1 cm. Close up of WT and TG spikelets at **g** 6 weeks and (**i**) 8 weeks after LD induction. Scale bar, 500 µm. Representative WT and TG floralets at **h** 6 weeks and **j** 8 weeks after LD induction. Scale bar, 500 µm. **k** Representative anthers of WT and TG plants. Scale bar, 100 µm. **l** Anther lengths of WT & transgenic turfgrass overexpressing Osa-miR396c were measured at the same stage after flowering. Data are shown as means (*n* = 6) with standard error. A significant difference between WT and TG anther length was indicated with asterisks at *P* < 0.001 by Student’s *t* test. **m** WT and **n** TG pollen was stained with potassium iodide. Scale bar, 100 µm

Creeping bentgrass is a cool season turfgrass, which requires vernalization for the competence to flower^25^. A previous study on colonial bentgrass (*Agrostis capillaris*), another species within the same genus as creeping bentgrass, revealed that 15 weeks of cold treatment [short-day (SD), 3–12 °C or LD, 3–6 °C] is required for inducing flowering^26^. To determine the length of cold treatment required to saturate the vernalization response in creeping bentgrass, two replicates of WT and *Os-miR396c* transgenic creeping bentgrass grown under SD conditions (14-h light, 25 °C/10-h dark, 17 °C) were subjected to cold exposure (8-h light/16-h dark, 5 °C) for 0, 11, 12, 13, 14, 15, 16, 17, and 18 weeks followed by shifting plants to LD conditions (16-h light, 25 °C/8-h dark, 17 °C). The results showed that WT plants do not flower under LD conditions until 15-week-cold exposure, whereas TG plants flower under LD conditions with or without cold exposure (from 0 to 18 weeks), indicating that TG plants overexpressing *Os-miR396c* are able to bypass the vernalization requirement to flower (Fig. 1). However, both TG plants (with or without vernalization) and vernalized WT controls require 4 weeks of LD (16-h light/8-h dark) induction for inflorescence emergence (Fig. 2a, d). The longer cold treatment of 18 weeks does not accelerate flowering in WT plants.

We then investigated the effect of photoperiod on flowering time in both WT and miR396 TG plants and found that neither TG plants without vernalization nor vernalized WT plants can flower under SD (14 h light) conditions. However, inflorescence emergence can be observed 4 or 3 weeks after a photoperiod of 16 or 24 h, indicating that overexpression of miR396 does not affect plant response to photoperiod. Both WT and miR396 TG plants are sensitive to photoperiod, and longer photoperiod leads to more rapid flowering (Supplementary Fig. S1).

Since miR396 is a conserved miRNA existing in both monocot and dicot species, we also investigated whether miR396-mediated elimination of vernalization requirement can be applied to dicot perennial species. Transgenic *C. flexuosa* overexpressing miR396 did not flower without vernalization treatment (Supplementary Fig. S3). The result indicates that the conserved miRNA also has species-specific functions.

### Overexpression of miR396c alters flower development that is associated with modified expression of target genes GRFs

To further investigate the impact of miR396 on creeping bentgrass reproductive growth, we compared floral organ development between WT and TG plants. During florescence emergence (4 weeks in LD) and anthesis (6 weeks in LD), the spikes of transgenic plants are curly in comparison with those of the WT controls (Fig. 2a, b, d, e). Two weeks later, the panicles of the WT plants become reddish to purple, while those of the miR396 transgenics remain green (Fig. 2c, f).

Microscopic analysis of the floralets in WT and TG plants during anthesis reveals that at the 6th and the 8th week in LD, miR396 TG plants exhibit defects in filament elongation and dehiscence, i.e., the filaments of the TG plants are shorter than those of the WT controls and the TG anthers do not normally dehisce (Fig. 2g–j). Statistical analysis of the anther length indicates that TG anthers are significantly longer than those of the WT controls (Fig. 2k, l).

Next, we examined pollen viability from TG and WT plants. As shown in Fig. 2m, n, WT pollens are circular and darkly stained (Fig. 2n), whereas TG pollens have varied shapes and are only lightly stained (Fig. 2n), indicating that pollens of transgenic plants are sterile.

Accumulating evidence indicates that miR396 plays an important role in floral organ development by post-transcriptionally repressing the expression of *GRFs*^19,20^. Seven of the nine *GRF* family members in *Arabidopsis* and ten of the twelve *GRFs* in rice have miR396 target sites^27^. In creeping bentgrass, we identified four *GRF* orthologs with miR396 binding sites. They are *AsGRF3*, *AsGRF4*, *AsGRF5*, and *AsGRF6*^22^. Their expressions are all repressed in miR396 TG plants compared with those in WT controls^22^, suggesting the participation of *GRFs* in modulating flower development in creeping bentgrass.

### MiR396 is induced by LD photoperiod and vernalization

**Fig. 3:**
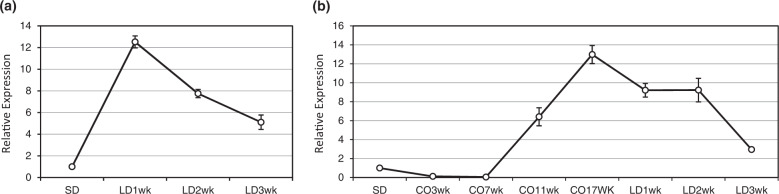
Expression profiles of miR396c under SD-LD, and SD-cold-LD conditions. **a** Stem-loop RT-qPCR analysis of miR396 expression in WT plants under SD conditions and in LD for 1–3 weeks without vernalization. **b** Stem-loop RT-qPCR analysis of miR396 expression in WT plants in SD, then 17 weeks of cold treatment, and under LD conditions for 3 weeks. The relative changes in gene expression were calculated based on the 2^−∆∆CT^ method. *AsUBQ* was used as an endogenous control. Data are presented as means of three technical replicates, and error bars represent ±SE

To decipher the molecular mechanisms underlying the miR396-mediated plant response to vernalization, we first examined how miR396 is regulated by different photoperiods and temperatures. To this end, we analyzed the expression levels of miR396 in WT plants under SD followed by transferring them to LD (referred hereafter as SD-LD) and exposure to prolonged cold followed by LD (referred hereafter as SD-cold-LD) conditions. When switching from SD (14-h photoperiod) to LD (16-h photoperiod), the abundance of miR396 was significantly elevated (12 folds) after 1 week in LD, and then gradually declined, but remained higher than that under SD conditions (Fig. 3a). When switching from SD to cold conditions, levels of miR396 first slightly declined at 3rd and 7th week, and then significantly induced, attaining 12 folds more than in SD 17 weeks after cold treatment (Fig. 3b). When shifting to LD conditions (25 °C at daytime/17 °C at night), levels of miR396 gradually declined but remained significantly higher than that under SD conditions (Fig. 3b). These results indicate that miR396 responds to and is induced by LD light regime and low temperature, suggesting its possible involvement in regulating plant response to these environmental cues.

### Effects of vernalization and different photoperiod regimes on VRNs in WT and TG creeping bentgrass

In winter cereals, *VRN1*, *VRN2*, and *VRN3* are key genes in the vernalization process for accelerating flowering^4,5^. This raises the question of whether or not in creeping bentgrass, miR396 affects plant vernalization response through regulating *VRN* gene expression. To address this question, we cloned the full-length orthologs of *VRN1*, *VRN2*, and *VRN3* (Supplementary Fig. S2) and compared their expression profiles under SD-LD and SD-cold-LD conditions in WT and TG creeping bentgrass.

**Fig. 4:**
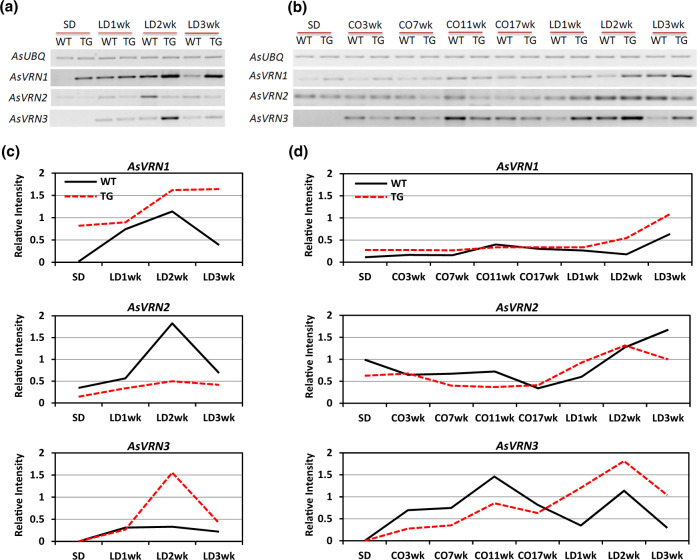
Expression profiles of *AsVRN1*, *AsVRN2*, and *AsVRN3* in SD-LD and SD-cold-LD conditions. **a** Semi-quantitative RT-PCR analysis of *AsVRN1, AsVRN2, and AsVRN3* gene expression in WT and TG plants under SD-LD conditions without vernalization. **b** Semi-quantitative RT-PCR analysis of *AsVRN1, AsVRN2, and AsVRN3* gene expression in WT and TG plants under SD-cold-LD conditions. *AsUBQ* was used as an endogenous control. Analysis of band intensity on electrophoresis gel is presented as relative ratios of *AsVRN1, AsVRN2, and AsVRN3* to *AsUBQ* under **c** SD-LD conditions and **d** SD-cold-LD conditions. The band intensity was quantified using ImageJ

Without vernalization, *AsVRN1* expression in WT plants is low under SD, but significantly induced when shifted to LD during the first 2 weeks. Its expression declines at LD 3-week, but remains elevated compared to that under SD (Fig. 4a, c), consistent with the expression profile of *VRN1* in wheat^28^. In miR396 transgenic creeping bentgrass, *AsVRN1* is induced in LD and remains elevated at LD 3-week (Fig. 4a, c). Interestingly, levels of *AsVRN1* are higher in TG plants than in WT controls under both SD and LD conditions (Fig. 4a, c), implying that *VRN1* is affected by miR396. During prolonged cold treatment, levels of *AsVRN1* are gradually increased and remain elevated following cold treatment in LD (Fig. 4b, d), which is in agreement with previous studies in cereals wheat, barley, and *Brachypodium*^12,29^. *AsVRN1* in TG plants has a similar expression profile to that in WT controls, though its levels are higher under SD and LD than that in WT plants, but not at the saturated cold (cold 17-week; Fig. 4b, d).

In barley, levels of *HvVRN2* (*HvZCCT-Ha* and *HvZCCT-Hb*) expression are higher when plants are grown in LD than in SD^30^. Similarly, in creeping bentgrass, levels of *AsVRN2* expression in WT plants increase significantly during the first 2 weeks of LD induction, and then decline at LD 3-week (Fig. 4a, c). In contrast, levels of *AsVRN2* in transgenic plants only slightly increase when switched from SD to LD (Fig. 4a, c), and compared with WT controls, *AsVRN2* in TG plants is repressed under both SD and LD conditions (Fig. 4a, c), suggesting that levels of *AsVRN2* expression are affected by miR396. It has previously been reported that during vernalization, *VRN2* transcript levels in both wheat and barley leaves decrease^7,30^. Consistent with this, *AsVRN2* expression in both WT and TG plants also gradually declines during vernalization, and then is elevated under LD conditions (Fig. 4b, d).

In wheat and barley, *VRN3* transcription is very low in SD and its expression is induced when plants are grown in LD^8,31^. In WT creeping bentgrass, *VRN3* exhibits a similar expression pattern to that in wheat and barley, i.e., *VRN3* is slightly induced when shifted from SD to LD (Fig. 4a, c). In contrast, TG plants overexpressing miR396 exhibit significantly enhanced *VRN3* expression at LD 2-week, and their *VRN3* expression then declines at LD 3-week (Fig. 4a, c). The dramatic upregulation of the flowering-promoting factor, *VRN3* suggests the transition of miR396 TG plants from vegetative to reproductive growth at LD 2-week. Besides the impact of day length on *VRN3* expression, vernalization has also been reported to upregulate *VRN3* in barley, wheat, and *Brachypodium*^8,32^. In accordance with this, *AsVRN3* mRNA levels are elevated during prolonged cold and the following LD treatments in both WT and TG plants, indicating that *AsVRN3* plays a role analogous to *VRN3* in wheat, barley and *Brachypodium*.

### Impacts of miR396 on chromatin structure modulating genes

**Fig. 5:**
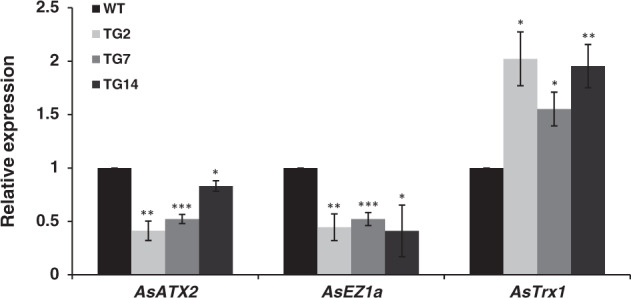
Expression levels of methyltransferases *AsATX2*, *AsEZ1a*, and *AsTrx1* in WT and TG plants under SD conditions. Real-time RT-PCR analysis of *AsATX2*, *AsEZ1a*, and *AsTrx1* expression in WT and three transgenic plants under SD conditions. *AsUBQ* was used as an endogenous control. Data are presented as means of three technical replicates, and error bars represent ±SE. A significant difference between WT and each TG lines at *P*<0.05, 0.01 or 0.001 by Student’s t-test was represented as "*", "**" or "***", respectively

Different regulations of *VRNs* by photoperiod between WT and miR396 TG plants without vernalization indicate that miR396 plays a role in the vernalization pathway. Since VRN1 is an upstream regulator in the vernalization pathway, we speculate that *VRN1* is directly or indirectly regulated by miR396. The upregulation of *HvVRN1* during vernalization has been reported to be associated with an increase of active histone marks H3K4 trimethylation (H3K4me3) and a decrease of silent marks H3K27me3 at *HvVRN1* chromatin, while levels of *VRN2* and *VRN3* in barley are not altered by histone modifications^9^. Therefore, it is hypothesized that *AsVRN1* might also be regulated by histone modifications during vernalization. In this study, higher levels of *AsVRN1* have been observed in TG plants than in WT controls under SD and LD without vernalization, which prompts us to examine if the levels of active and silent histone marks are different in WT and TG plants. Figure 5 shows that *AsATX2*^33–36^ and *AsTrx1*^36–39^, the two trithorax group (*TrxG*) genes encoding homologs to *Drosophila* trithorax (Trx) that mediate methylation at H3K4 are down- and up-regulated in three transgenic lines, respectively. *AsEZ1a*^34,36,40,41^, a polycomb group (*PcG*) gene encoding a homolog to the Enhancer of Zeste [E(z)] that mediates methylation at H3K27 is repressed in TG plants. The results imply that miR396 might contribute to histone methylation to modulate chromatin structure regulating the expression of *VRNs*.

### Effects of day length and vernalization on GRFs in WT and TG plants

**Fig. 6:**
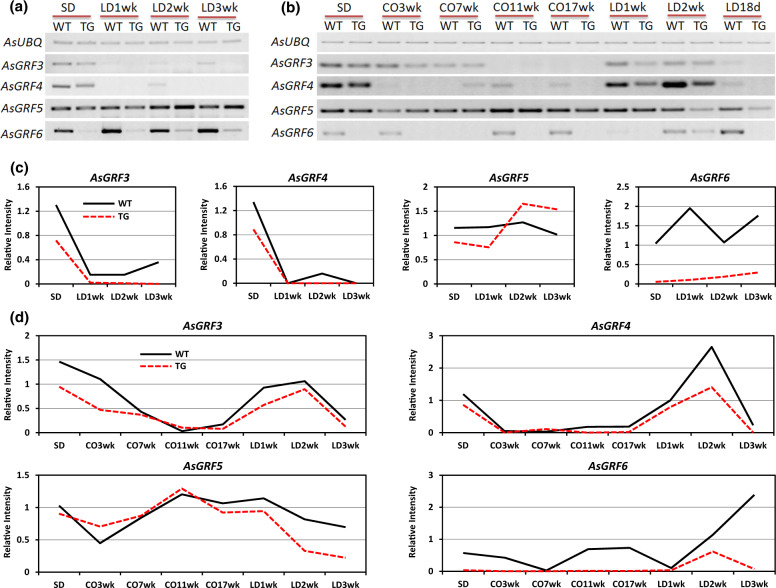
Expression profiles of the putative miR396c targets *AsGRF3, AsGRF4, AsGRF5*, and *AsGRF6 in* SD-LD and SD-cold-LD conditions. **a** Semi-quantitative RT-PCR analysis of *AsGRF3, AsGRF4, AsGRF5, and AsGRF6* gene expression profiles in WT and TG plants under SD-LD and **b** SD-cold-LD conditions. **c** Analyses of band intensity on electrophoresis gel are presented as relative ratio of *AsGRF3, AsGRF4, AsGRF5, and AsGRF6* to *AsUBQ* under SD-LD conditions and **d** SD-cold-LD conditions. The band intensity was quantified using ImageJ

Transcript levels of miR396 putative targets were also analyzed under SD-LD and SD-cold-LD conditions in both WT and TG plants. MiR396 is significantly induced and remains elevated when plants are transferred from SD to LD (Fig. 3a). In contrast, levels of its targets, *AsGRF3* and *AsGRF4* are significantly down regulated when switching from SD to LD conditions in both WT and TG creeping bentgrass (Fig. 6a, c). Interestingly, expression profiles of *AsGRF5* and *AsGRF6* in WT plants do not show negative correlation with the levels of miR396 in SD-LD, suggesting that *AsGRF5* and *AsGRF6* might also be regulated by light-related factors besides their direct repressor miR396. In addition, levels of *AsGRF5* are higher in transgenics than in WT controls under LD induction (Fig. 6a, c), suggesting that miR396 might impact *AsGRF5* by regulating other unknown factors.

### Gene ontology enrichment analysis in miR396 TG plants

To further understand how miR396 is involved in floral organ development and flowering time control, we analyzed the previously obtained RNA-seq data to study the differentially expressed genes (DEGs) in miR396 TG plants vs. WT controls^22^. The WT and mR396 TG cDNA libraries had been prepared using RNA extracted from the shoot samples of the LD 3-week (without vernalization) plants for Illumina sequencing^22^.

**Fig. 7:**
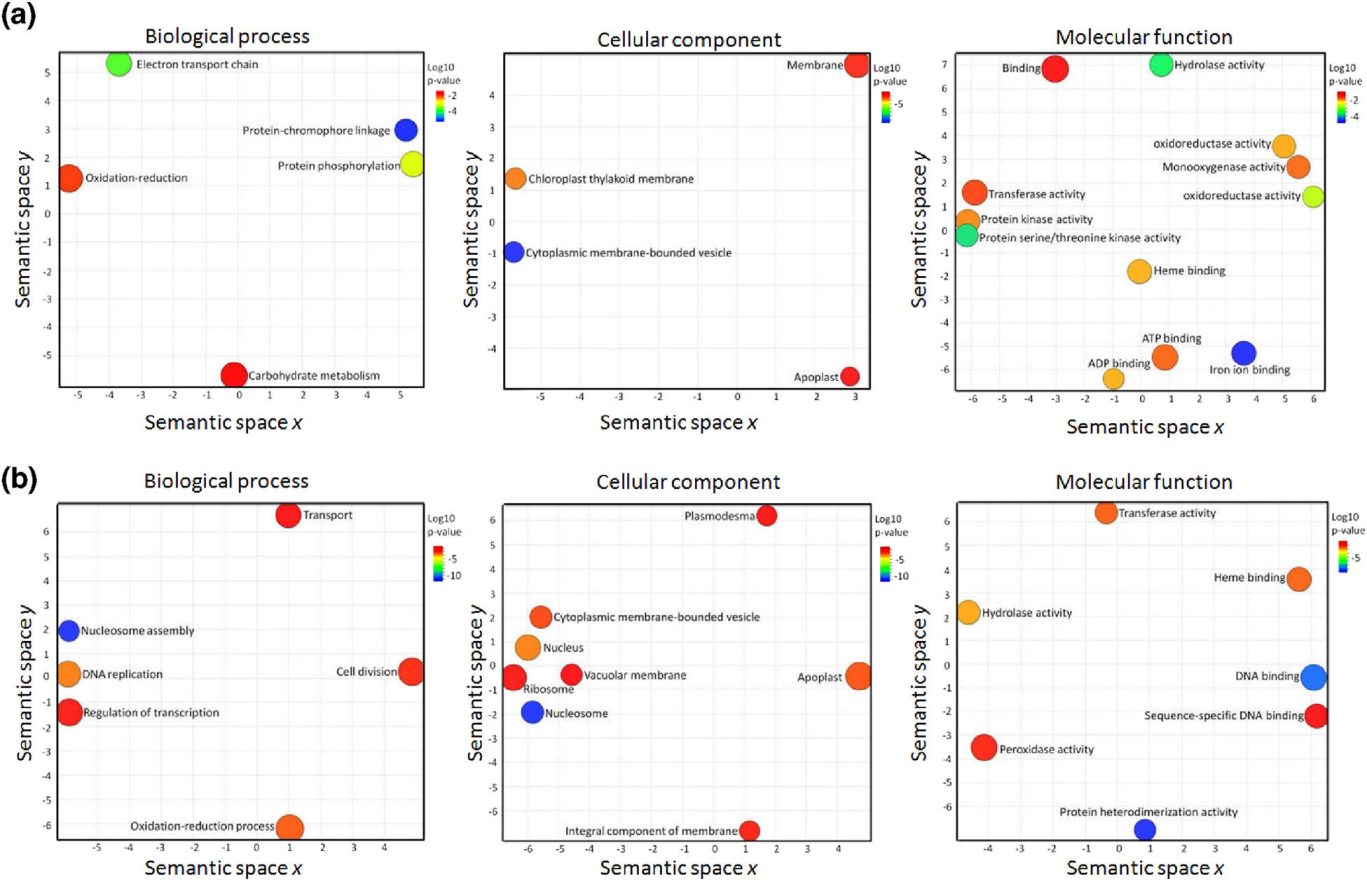
GO enrichment analysis. Significantly enriched GO terms for genes **a** up-regulated (log_2_ FC > 2) and **b** down-regulated (log_2_ FC < -2) in TG vs. WT are projected onto a two-dimensional semantic space with three GO categories, including biological process, cellular component, and molecular function. Each bubble represents a GO term. The closer the bubbles rest, the more related the GO terms are. The bubble color represents the significance of the enrichment. Bubbles with more general GO terms are larger

To identify the major functional categories, which are represented in transgenics vs. WT controls, we performed gene ontology (GO) enrichment analysis. Figure 7 shows that 21 GO terms were significantly enriched (over-represented *p*-value < 0.05) in up-regulated (log_2_ FC >2) and down-regulated (log_2_ FC <−2) genes, respectively. Among others, the GO terms ‘electron transport chain’, ‘carbohydrate metabolism’, ‘chloroplast thylakoid membrane’, and ‘ATP binding’ are enriched in the up-regulated genes. Under LD 3-week induction without vernalization, TG plants are in the flower development stage while WT plants are still in vegetative growth. These enriched GO terms are related to energy generation and metabolism, which are fundamental for energy supply, carbon storage, and cell wall formation during flower development. This result is in close agreement with a study in other plant species during flower development^42^. The enriched GO terms in the down-regulated genes include ‘cell division’, ‘DNA replication’, ‘regulation of transcription’, ‘nucleus’, and ‘DNA binding’, indicating that the processes of DNA replication and cell division are strongly repressed in TG plants overexpressing miR396. This result is consistent with the miR396-GRF system, which shows decreased cell number in miR396 transgenic leaves through repressing levels of *GRFs*^15^.

### Differential expression of transcription factor genes

**Fig. 8:**
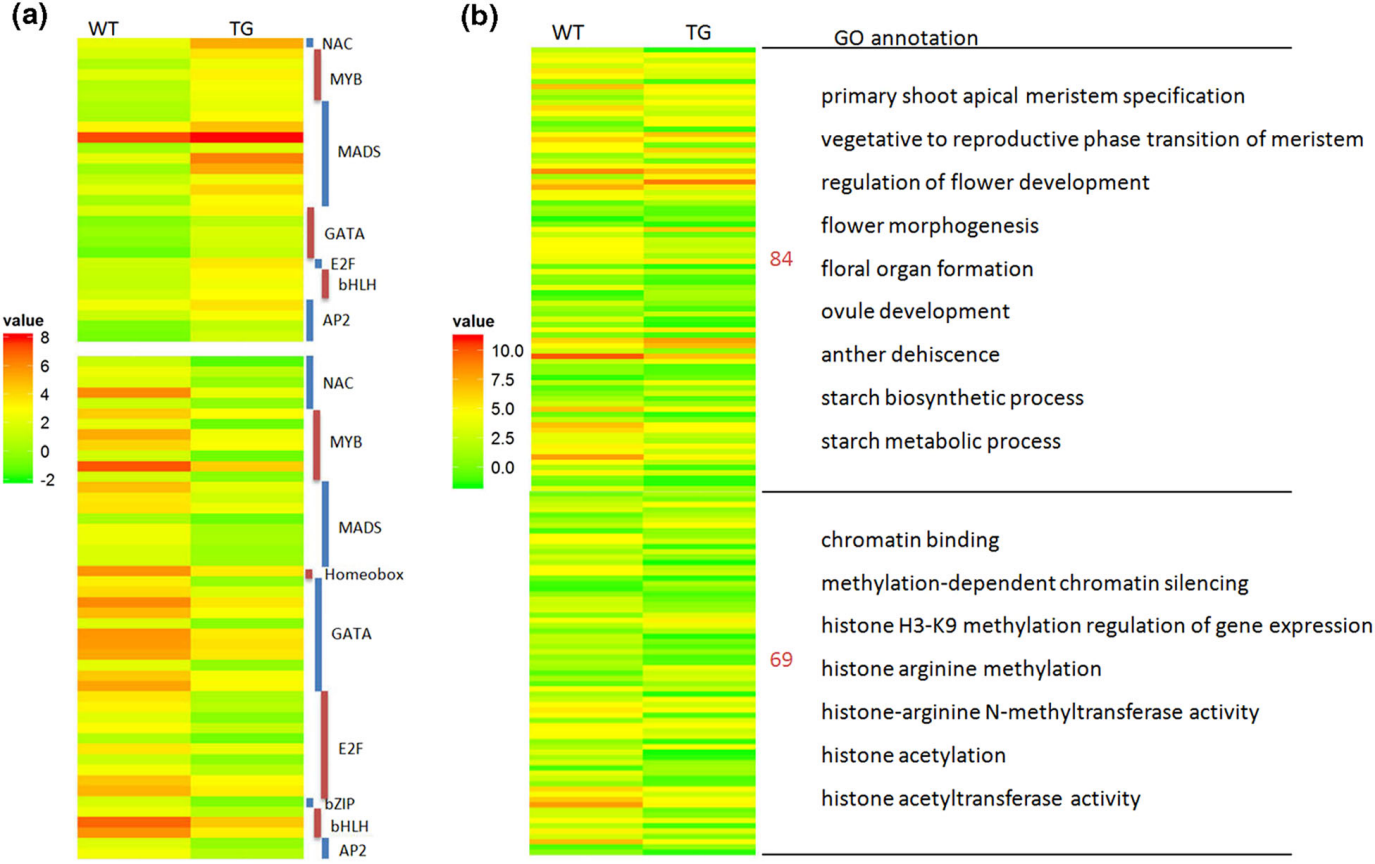
Differential expression analysis. **a** Differential analysis of transcription factor genes at 3-week LD induction. Various transcription factor families with differential expression (log_2_ FC > 1 and log_2_ FC < −1 in upper and lower panel, respectively) are shown in the heatmap. **b** Differential expression of flower development and chromatin modification-related genes. Significantly enriched GO terms (log_2_ FC > 1 or log_2_ FC < −1, FDR < 0.05), which relate to flower development and chromatin modification, in TG vs. WT data sets are listed. The corresponding differentially expressed genes (DEGs) of each enriched GO term were selected to generate the heatmap. The color gradient shows the log_2_-transformation of the read count value

MiRNAs are involved in various plant physiological processes through regulating their targets, most of which are transcription factors (TFs). In addition, many TFs are key regulators implicated in flowering time control and flower development. In this study, 77 genes from 9 TF families are differentially expressed (log_2_ FC >1 or <−1, FDR corrected *p*-value < 0.05) in TG vs. WT plants, including NAC, MYB, MADS, GATA, E2F, bHLH, AP2, homeobox, and bZIP (Fig. 8a). Among them, the MADS-box TF family, which consists of 10 up-regulated and 8 down-regulated members, is most represented. The second most highly represented is GATA family (5 up-regulated and 11 down-regulated) followed by MYB TF genes (5 up-regulated and 7 down-regulated).

MADS-box TFs play an essential role during plant flowering, which includes transition to flowering, petal and stamen specification, carpel and ovule development, pollen maturation and tube growth, and sepal and petal longevity^43^. Many GATA family members play a predominant role in floral development. For example, *GNC* and *GNL* from GATAs are flowering repressors through regulating the expression of florigen *SOC1*, while *HAN* serves as a floral morphology regulator through controlling the homeobox TF *WUSCHEL*^44^. MYB TFs are critical for floral asymmetry^45^.

### Differential expression of flower development and chromatin modification genes

Besides identifying the major functional categories over-represented in transgenics, we are also interested in the flower development and chromatin modification-related genes in TG vs. WT plants. The later may play a role in epigenetically regulating *VRN* gene expression to control flowering. Therefore, we determined the significantly enriched GO terms (over-represented *p*-value < 0.05), which relate to flower development and chromatin modifications. The corresponding differentially expressed genes (DEGs, log_2_ FC >1 or log_2_ FC <−1, FDR corrected *p*-value < 0.05) from each GO term were selected to generate a heatmap (Fig. 8b). Figure 8b shows that 84 genes were categorized into the flower development group, which includes processes of SAM specification, vegetative to reproductive transition, flower organ formation, anther dehiscence, and starch biosynthesis and metabolism; 69 genes are categorized into the chromatin modification group, including the processes of histone acetylation and methylation. These results provide evidence at the molecular level about the differences in reproductive transition and development we observed between TG plants and WT controls, such as altered flowering time, anther dehiscence defect, pollen sterility, and different levels of *VRN1*.

## Discussion

### MiR396-mediated flower development

MiR396 regulates plant leaf growth and floral organ development through targeting *GRFs*. Overexpression of miR396 causes distinct floral organ defects in different plant species, such as open husks and long sterile lemmas, abnormal pistils, altered anther and carpel morphology in transgenic rice, *Arabidopsis*, and tobacco^18–20^. In this study, transgenic creeping bentgrass plants display abnormal stamen development, which includes reduced filament extension, dehiscence defects, and pollen sterility. It is likely that *GRF* members from different plant species target different genes in SAMs to regulate floral organ development, thereby leading to distinct flower phenotypes. In transgenic rice overexpressing *OsmiR396d*, for example, altered floral organ morphology was shown to result from the repressed expression of *OsGRF6* and *OsGRF10*, which targets a H3K9 demethylase gene *OsJMJ706* and a kinase gene *OsCR4* involved in the maintenance of the palea and lemma interlocking^20^. However, the regulatory networks between other GRF homologs and downstream targets in rice and other plant species remain unknown. Further identification of GRFs’ targets in different plant species would provide information to better understand miR396-GRF module-mediated floral organ development.

Plant male sterility provides benefits not only in preventing pollen-mediated gene flow in transgenic plants, but also in blocking self-fertilization facilitating hybrid production for new breeding opportunities. Overexpression of *Os-miR396c* in creeping bentgrass resulted in male sterility with plant defects of short filaments, indehiscent anthers, and immature pollen grains. Interestingly, the similar male sterile phenotype was also observed in transgenic *Arabidopsis* with repressed expression of a bHLH TF gene *MYC5* through overexpression of a MYC5-SRDX chimeric repressor, and in transgenic tobacco with ectopic expression of a soybean MADS-box TF gene, *GmMADS28*^46,47^. *MYB26* is responsible for anther dehiscence through regulating *NST1* and *NST2* from NAC TF family^48,49^. The TF families mentioned above are differentially expressed in miR396c TG plants and WT controls, implying that miR396 functions in stamen development through regulating other genes, especially TF genes. It is also plausible that the male sterile phenotype may be directly attributed to miR396-GRF module.

In the present study, the lemmas of WT spikelets turn reddish-brown when floral organs begin to senesce, whereas miR396 transgenic spikelets remain green (Fig. 2i, j). The reddish-brown color in the spikelet results from the synthesis of anthocyanins and the degradation of chlorophyll during plant senescence. Given that oxidative stress triggers plant senescence, we speculate that overexpression of miR396 may have delayed flower senescence in transgenic plants by enhancing plant tolerance to oxidative stress. Our hypothesis is further supported by the result of GO enrichment analysis, in which the GO terms oxidation-reduction and oxidoreductase activity are enriched in up- and down-regulated genes (Fig. 7). However, previous studies show that high levels of *GRF3* and *GRF5* delay leaf senescence in transgenic *Arabidopsis*^50,51^, implying that constitutive expression of miR396 might trigger, other than delay, leaf senescence. The controversial results could be attributed to the species-specific function of miR396-GRF module. In addition to the direct regulation of plant senescence via the miR396-GRF pathway, differential regulation of anthocyanins accumulation in WT and TG plants is another possibility. TF families MYB and bHLH MYC are implicated in the process of anthocyanins synthesis^52^. Since MYB and bHLH TF families show differential expression in transgenics and WT controls as shown in Fig. 8a, it is likely that the anthocyanins synthesis pathway is affected in transgenics overexpressing miR396, which leads to the green spikelets in TG plants.

### Elimination of vernalization requirement

In temperate climates, flowering time must coincide with optimal environmental conditions for reproductive success. Many plants sense and respond to prolonged winter cold and flower next spring when day length increases. However, winter temperature varies from year to year and from place to place, which largely limits agricultural practices. Genetic modifications of plants to eliminate vernalization requirement overcome these constraints and expand regions for agricultural practices. In addition, flexible flowering time is an advantage in reducing damages from certain environmental stresses. Furthermore, effective elimination of vernalization allows biennials and perennials to flower in the first year, which can be applied to accelerate the introgression of new agronomic traits in breeding process.

Currently, the elimination of the vernalization requirement has been accomplished through genetic engineering of vernalization pathway genes in both dicot and monocot species. In *Arabidopsis*, *FRIGIDA* (*FRI)* complex positively regulates *FLC* to repress flowering^53^. Evidence shows that the vernalization requirement is eliminated in plants containing mutations in the components of the complex, such as *FRI*, *SUPPRESSOR OF FRI4*, *FRI ESSENTIAL1*, and *FLC EXPRESSOR*^54–56^. A similar result is also obtained in wheat with deletions or mutations in the monocot flowering repressor *VRN2*^4^. Besides the flowering repressor, modification of the flowering activator *VRN1* leads to the same phenotype. The wheat gene with a mutation in the *VRN1* promoter or large deletions in its first intron could bypass the vernalization requirement^57^. The same phenotype could also be achieved through activating flower integrator. In Medicago, vernalization and the following LD conditions induce the *FT* ortholog *FT1a*. A gain-of-function mutant with high levels of *FT1a* transcripts exhibits up-regulated *SOC1* and *FULb*, resulting in early flowering without vernalization requirement in LD^58^. In this study, levels of the vernalization pathway genes were altered in miR396 TG creeping bentgrass, suggesting that the elimination of vernalization is accomplished through regulating *VRNs*. In SD-LD conditions, the flowering activator *VRN1* remained elevated, while the flowering repressor *VRN2* remained repressed in TG plants. Based on the vernalization pathway established in wheat and barley, we conclude that high levels of VRN1 repress *VRN2* expression. In comparison with WT controls, TG plants displayed significantly up-regulated *VRN3* expression in LD when switched from SD conditions. This might result from the repression of *VRN2* expression, thereby promoting flowering of TG plants bypassing vernalization requirement.

A miRNA-mediated early flowering phenotype has been observed through regulating flowering integrators, such as *SOC1* and *FT*. For example, the *Pooideae*-specific miRNA, miR5200 targets *FT* mRNA in *Brachypodium*^59^. Artificial interruption of miR5200 in SD leads to early flowering^59^. In the biennial-to-perennial plant *C. flexuosa*, the miR156-*SPL*s-miR172-*AP2* module regulates *SOC1* to control flowering time^12^. Wild-type *C. flexuosa* does not respond to vernalization until plants are 5-week-old. Artificial interruption of miR156 or overexpression of miR172 results in an early response to cold or even the elimination of the vernalization requirement. In this study, we speculate that miR396 may be critical for the elimination of vernalization through regulating vernalization pathway in creeping bentgrass, although the mechanisms behind this remain unknown. Stem-loop RT-qPCR analysis shows that miR396 in WT plants was induced in LD and prolonged cold conditions. Thus, it is hypothesized that prolonged cold leads to the upregulation of miR396, which then turns unvernalized plants to the vernalized state through modulating its direct target(s) or other unknown regulatory element(s), and then LD induces floral homeotic genes in the “ABC” model to specify floral organs. High levels of miR396 in TG plants result in the “vernalized state” without cold treatment, and then flowering after LD induction. It should be noted that there is a discrepancy in correlated expression of miR396 and *VRN* genes during vernalization. The miR396 expression gradually increases and peaks at 17 weeks of vernalization treatment (Fig. 3b), but the expression profiles of the *VRN* genes (Fig. 4b, d) do not seem to correlate as well as we would expect. Most likely, this is because miR396 is not the only factor determining the expression of *VRN* genes. During vernalization process, miR396 might be gradually up-regulated and function to activate epigenetic regulation mechanism to modulate *VRN* gene expression. However, miR396-mediated *VRN* regulation might be inefficient until after the vernalized plants are shifted to LD conditions, which would fully trigger miR396-mediated *VRN* regulation, leading to significant change in *VRN* expression, and therefore plant flowering. Further research is needed to provide direct experimental evidence validating the role miR396 may play in activating epigenetic regulation mechanism to modulate *VRN* gene expression.

In this study, overexpression of miR396 in a dicot species, *C. flexuosa*, did not lead to flowering in the unvernalized transgenic plants (Fig. S3) indicating that the conserved miRNA also has species-specific functions. Given that monocot and dicot species establish their vernalization requirement by recruiting different genes, it is not surprising that miR396 is not involved in vernalization pathways of this dicot species. Further analysis in perennial monocot species would reveal whether the role of miR396 in flowering time control is conserved.

### Histone modifications and VRN1

Histone modifications in response to vernalization have been well studied in *Arabidopsis*. Vernalization leads to the stable repression of *FLC* through an increase in silent marks of H3K27me2, H3K27me3, H3K9me2, and H4 arginine 3 dimethylation, and a decrease in active marks of H3K4me2, H3K4me3, and histone acetylation on *FLC* chromatin^60,61^. H3K4 methylation at *FLC* chromatin was shown to be catalyzed by two TrxG proteins, ARABIDOPSIS TRITHORAX-LIKE 1 (ATX1) and ATX2 homologous to the *Drosophila* H3K4 methyltransferase^33–38^. Similarly, the rice *Oryza sativa Trithorax1* (*OsTrx1*) gene encoding a TrxG protein also exhibits histone H3 methyltransferase activity and is involved in regulating flowering time by modulating chromatin structure in rice^39^. H3K27 methylation at *Arabidopsis FLC* chromatin was shown to be mediated by CURLY LEAF (CLF), a homolog of the *Drosophila* histone methyltransferase E(z), which is a PcG protein^34,36,40^. Similarly, a homolog of *CLF/ENHANCER OF ZESTE like*‐*1* (*EZL1*) in *Brachypodium distachyon* was also demonstrated to regulate plant flowering by modulating H3K27me3 at the chromatins of the flowering genes *VRN1* and *AGAMOUS* (*AG*)^41^.

In winter wheat and barley, initiation of reproductive development is mediated by stable induction of *VRN1*^9,62^. A previous study indicates that vernalization results in the changes of *VRN1* chromatin state with the increased levels of active marks H3K4me3 and decreased levels of silent marks H3K27me3 across the 5′ end of the barley *VRN1* (*HvVRN1*) gene, which includes regions within the 2-kb promoter and regions associated with regulation of *HvVRN1* expression within the 10.8-kb first intron^9^. In this study, elevated levels of *AsVRN1* in miR396 TG plants prompted us to examine the transcript levels of H3K4 and H3K27 methyltransferases in TG vs. WT plants. To this end, we analyzed the expression of *AsATX2* and *AsTrx1*, the creeping bentgrass homologs of the *Drosophila* H3K4 methyltransferase *Trx* gene, and *AsEZ1a*, a creeping bentgrass homolog of the *Drosophila* H3K27 methyltransferase *E(z)* gene. Quantitative RT-PCR analysis shows that H3K27 methyltransferase gene *AsEZ1a* is repressed in transgenic lines, which might release the repression on *AsVRN1* chromatin. Interestingly, levels of two H3K4 methyltransferase genes *AsATX2* and *AsTrx1* are down- and up-regulated in three transgenic lines compared to WT controls. Thus, the hypothesis that *AsVRN1* in TG plants is induced through the elevated active marks of H3K4 methyltransferases cannot be confirmed. It is likely that the chromatin state of other genes is also affected in miR396 TG plants. Therefore, the validated conclusion cannot be made through analyzing the transcript levels of H3K4 and H3K27 methyltransferase genes. Direct analysis on the relative abundance of H3K4me3 and H3K27me3 would demonstrate the histone modification state of *AsVRN1*. Unfortunately, due to the unavailable genomic information in creeping bentgrass, ChIP-seq analysis for examining the relative abundance of histone modifications is difficult to conduct.

Among DEGs in TG vs. WT of this study, histone modification-related GO terms were significantly enriched, such as histone acetylation, histone H3K9 methylation regulation of gene expression, and histone arginine methylation. The result suggests that in addition to H3K27 and H3K4 trimethylation, creeping bentgrass responding to vernalization might recruit other mechanism of histone modification as what has been revealed in *Arabidopsis*.

Thus far, some evidence has shown that the miR396-GRF module might be involved in histone modifications. A previous study indicates that target GRFs contain a conserved QLQ (glutamine, leucine, and glutamine) motif, which is implicated in chromatin remodeling in the SWI2/SNF2 protein genes of *Saccharomyces cerevisiae*^63^. In addition, overexpression of the maize (*Z. mays*) *GRF* gene *ZmGRF10* leads to the down-regulation of several genes encoding chromatin-modifying proteins^64^. A GIF that forms a complex with GRF to regulate downstream transcription activities binds to various chromatin remodeling proteins as well^65^. A recent study indicates that a H3K9 demethylase gene *OsJMJ706* involved in floral organ development is regulated by the *OsmiR396d*-*OsGRF6*/*OsGRF10* module in rice^20^. It is plausible that the miR396-GRF module is also involved in vernalization pathway in creeping bentgrass or other cereals. Besides *GRFs*, *SHORT VEGETATIVE PHASE* (*SVP*) has been identified as a target of miR396 in *Arabidopsis*^66^. AtSVP is a MADS-box TF, which forms a complex with AtFLC to repress the expression of *AtFT* and thereby controls flowering time. To control the proper time for floral organ patterning, AtSVP also silences the chromatin of a floral organogenesis-related gene *SEPALLATA3* through modulating H3K27me3^14^. Thus, it is likely that miR396-SVP module is also involved in histone modification in cereals to control flowering time. Validation of SVP as a target of miR396 and investigation of its role in histone modification in creeping bentgrass or other cereals would provide evidence of miR396-mediated flowering time control.

In summary, this work shows that male sterility and plant response to vernalization are dependent on the miR396c-GRF pathway or other miR396c-mediated mechanisms in the perennial monocot species, creeping bentgrass. The male sterility trait may be of use for the genetic hybridization in breeding. Elimination of the vernalization requirement for flowering could provide benefits on expanding the geographic area for agriculture practice, avoiding unfavorable environments, and accelerating breeding process.

## Supplementary information

Supplemental Figures
