# Supplementary material for: MiR396 is involved in plant response to vernalization and flower development in Agrostis stolonifera
Source: Hortic Res. 2020 Nov 1;7:173. doi: 10.1038/s41438-020-00394-x (PMC7603517; doi:10.1038/s41438-020-00394-x)
Supplement: Supplementary file 1 — Supplemental Figures [file 41438_2020_394_MOESM1_ESM.docx]

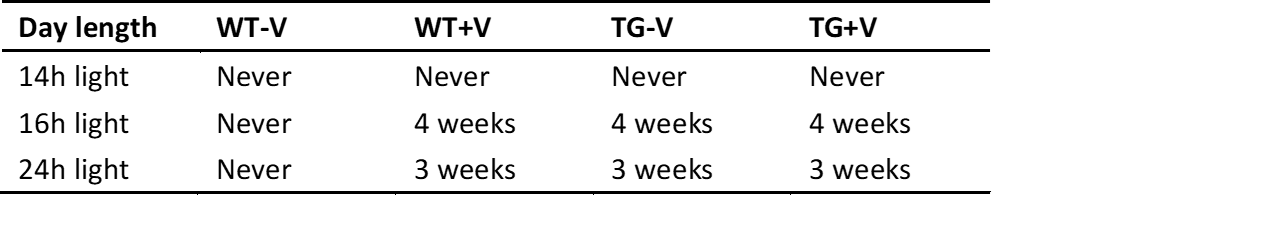


**Figure S1.** Impacts of day length on flowering in WT and TG creeping bentgrass. WT-V: WT creeping bentgrass without vernalization treatment; WT+V: WT plants with vernalization treatment; TG-V: *miR396c* transgenic creeping bentgrass without vernalization treatment; TG+V: *miR396c* transgenic plants with vernalization treatment.

**Figure S2.** Phylogenetic analyses of VRN1, VRN2, and VRN3 proteins and sequence alignment of their conserved domains. Phylogenetic trees of (a) VRN1, (b) VRN2, and (c) VRN3 from creeping bentgrass, Brachypodium, wheat, barley, rice and Arabidopsis were built with the neighbor-joining method. Bootstrap values were derived from 1000 replications. Alignment of (d) type II subfamily of MADS domain and K-box, (e) CCT domain, and (f) PEBP (phorsphatidylethanolamine) domain among different plant species, including creeping bentgrass, Brachypodium, wheat, barley, rice and Arabidopsis*.* Species abbreviations: As=*Agrostis stolonifera*, Bd=*Brachypodium distachyon*, Ta=*Triticum aestivum*, Tm=*Triticum monococcum*, Hv=*Hordeum vulgare*, Os=*Oryza sativa*, and At=*Arabidopsis thaliana*. Similarities of conserved domains between creeping bentgrass and each of the orthologs are listed at the end of the alignment.


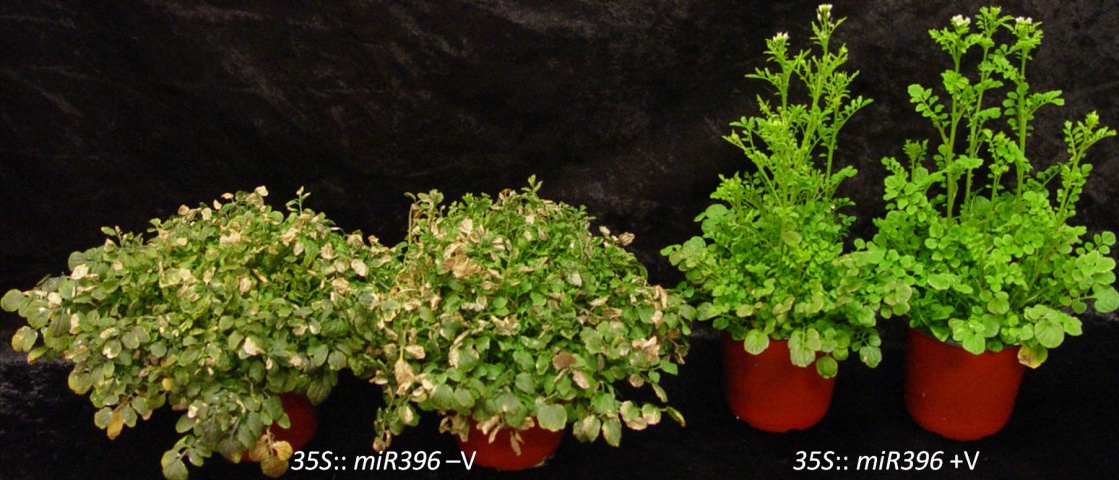


**Figure S3.** Vernalized (+V) and non-vernalized (-V) transgenic *C. flexuosa* overexpressing miR396 under 2-week LD induction. Non-vernalized TG *C. flexuosa* plants were not flowering after LD induction for 15 weeks, while 5-week-old TG plants were flowering after 8-week cold treatment followed by 2-week LD induction.

**Table S1.** Primer sequences used in this study.

| **Name** | **Sequence (5' - 3')** | **Purpose** |
| --- | --- | --- |
| ***AsUBQ* q-F** | GGCGTCATCGACCTTGTAGA | For q-PCR of *AsUBQ* |
| ***AsUBQ* q-R** | GACAACGTCAAGGCCAAGAT |  |
| ***miR396* q-F** | GCGGTTCCACAGCTT TCTT | For q-PCR of *miR396* |
| ***miR396* q-R** | TGGTGCAGGGTCCGAGGTATT |  |
| ***AsGRF3*-F** | CAAGAAAGCCTGTGGAAACG | PCR for mRNA of *AsGRF3* |
| ***AsGRF3*-R** | AGCGAGTGGTTCTGGAAAG |  |
| ***AsGRF4*-F** | ACTACCGCCTCTTCCCC | PCR for mRNA of *AsGRF4* |
| ***AsGRF4*-R** | CATTGCTACATGCTGAGAACG |  |
| ***AsGRF5*-F** | GTTAAAACCAATGGCCTGTCTC | PCR for mRNA of *AsGRF5* |
| ***AsGRF5*-R** | TGCTCCAGTCAAGAAACTCAG |  |
| ***AsGRF6*-F** | CCAGGCTGGAGAGTGTTTATG | PCR for mRNA of *AsGRF6* |
| ***AsGRF6*-R** | GCGGATGCTCGGATGATTTA |  |
| ***AsVRN1*-F** | CAAGATAAACCGACAGGTGACC | PCR for mRNA of *AsVRN1* |
| ***AsVRN1*-R** | GACCTCTCCTTCTTCTGTAGCTC |  |
| ***AsVRN2*-F** | GAGAAGCAGATCCGTTACGAG | PCR for mRNA of *AsVRN2* |
| ***AsVRN2*-R** | TCTTCATGTACCTTGGCGAAG |  |
| ***AsVRN3*-F** | ACCCATTCGTCCGTACCACAAA | PCR for mRNA of *AsVRN3* |
| ***AsVRN3*-R** | AGGTTGTAGAGCTCGGAGAAGT |  |
| ***AsATX2* q-F** | CCTGAAGGGTATACTGCTTTCAG | q-PCR for mRNA of *AsATX2* |
| ***AsATX2* q-R** | CAGATCTTTGACAAACGGTTCCT |  |
| ***AsEZ1a* q-F** | CCGCATCTGGAACACTTAGC | q-PCR for mRNA of *AsEZ1a* |
| ***AsEZ1a* q-R** | GAGACATGGACACTCTTTCCC |  |
| ***AsTrx1* q-F** | CATCTGAGGAAATGAAGTGCCTG | q-PCR for mRNA of *AsTrx1* |
| ***AsTrx1* q-R** | CGATTGATCTAACCGGACTGG |  |
